# Visual Harmony and Complexity Shape Subjective Judgments More than Detectable Overt Attention: Evidence from Eye-Tracking and Facial Coding

**DOI:** 10.3390/jemr19050105

**Published:** 2026-09-18

**Authors:** Horacio Rostro-Gonzalez, Ana M. S. Gonzalez-Acosta, Victor H. Jimenez-Arredondo

**Affiliations:** 1 Department of Electronics Engineering, DICIS–University of Guanajuato, Carretera Salamanca–Valle de Santiago, Salamanca 36885, Guanajuato, Mexico; 2 Department of Industrial Engineering, School of Engineering–IQS, Universitat Ramon Llull, Via Augusta, 08017 Barcelona, Spain; 3 Doctoral Program in Biosciences, Faculty of Medicine and Surgery, Benito Juárez Autonomous University of Oaxaca, Av. Universidad, Oaxaca 68120, Oaxaca, Mexico; goaa800904.fmc@uabjo.mx; 4 Department of Art and Business, DICIS–University of Guanajuato, Carretera Salamanca–Valle de Santiago, Salamanca 36885, Guanajuato, Mexico; vhjimenez@ugto.mx

**Keywords:** visual attention, eye tracking, visual complexity, scanpath entropy

## Abstract

Understanding how visual structure shapes attentional allocation is central to models of perceptual processing. Less is known about how formal properties like harmony and complexity shape exploration independent of salience or semantic content. This study examined how controlled structural variations relate to attention, facial engagement, and subjective judgment using eye-tracking and webcam-based facial coding. Participants (N=40) viewed stimuli derived from a common geometric base, manipulated into three conditions: high harmony (symmetrical, low complexity), high complexity (asymmetrical, disorganized), and structured complexity (high complexity with underlying order). Eye movements and facial expressions were recorded during free viewing. Metrics included time to first fixation, fixation duration, number of fixations, scanpath entropy, spatial dispersion, and facial-coding indices (neutral, happy, and surprise expression, and the ambient/focal coefficient *K*). None of the eye-tracking or facial-coding metrics differed significantly across conditions; given that the study was powered to detect only medium-to-large effects, this indicates no detectable difference under the present webcam-based, brief-exposure design rather than evidence that visual structure has no effect on attention. Subjective complexity ratings differed robustly across conditions, surviving correction for multiple comparisons: unexpectedly, the high-complexity condition was rated as less complex than the harmony and structured-complexity conditions, indicating the intended manipulation did not translate into perceived complexity as designed. A nominally significant difference in pleasantness ratings did not survive this correction. Using repeated-measures correlation to account for the non-independence of within-participant observations, scanpath entropy showed a nominal negative association with pleasantness that did not survive correction for multiple comparisons, and the coefficient *K* showed a weaker and partly inconsistent pattern of association with independent oculomotor indices than initial uncorrected analyses suggested. These findings indicate that, in the present study, formal visual structure shaped subjective complexity judgments more robustly than it shaped overt attentional or facial-affective engagement, a pattern consistent with—though not conclusive proof of—a broader dissociation between evaluative and attentional responses reported in face perception and developmental aesthetics research. Beyond this substantive finding, we report in detail how accounting for repeated-measures non-independence and applying an explicit multiplicity strategy changed our statistical conclusions, offering a worked methodological example for similarly structured webcam-based eye-tracking and facial-coding studies.

## 1. Introduction

Visual attention is guided by an interplay of bottom-up and top-down processes that determine how information is selected and processed within complex visual environments. Classic models of attentional selection have emphasized the role of low-level salience—such as contrast, color, and orientation—in capturing attention [1], as well as the influence of task demands and prior knowledge in guiding gaze behavior [2]. However, beyond these well-established factors, there is increasing interest in understanding how higher-order visual structure modulates attentional allocation in the absence of semantic content.

From a perceptual perspective, Gestalt principles such as symmetry, proximity, and continuity provide a framework for understanding how the visual system organizes information into coherent structures [3]. Symmetry in particular occupies a privileged position in this literature: symmetrical configurations are judged as more aesthetically pleasing than their asymmetrical counterparts across a wide range of stimuli [4], and classic eye-tracking work has shown that symmetry systematically reorganizes visual scanning, with fixations for symmetrical shapes clustering non-uniformly across the figure while both fixation count and fixation time scale directly with structural complexity [5]. This symmetry preference generalizes across a heterogeneous range of stimulus categories, including abstract shapes, faces, and natural scenes, although its strength varies considerably by category [6], and more recent eye-tracking work indicates that observers spontaneously scan along a pattern’s axis of reflection in a manner that is similar whether they are explicitly classifying or evaluating the stimulus, suggesting that this oculomotor signature of symmetry detection is not simply a byproduct of an evaluative task [7]. At the same time, whether symmetry detection is itself an attentionally demanding process, or occurs preattentively, remains debated [8], suggesting that the relationship between structural regularity and the deployment of visual attention is more nuanced than a simple facilitation account would predict. Recent studies using eye tracking have demonstrated that compositional structure more broadly influences gaze distribution and fixation patterns, highlighting the role of visual organization in guiding attention [9,10,11].

Visual complexity has also been shown to systematically modulate attentional behavior. Increasing levels of complexity are associated with higher cognitive load and changes in fixation patterns, often resulting in more focal and localized attentional allocation [12]. Modeling what makes an image look complex in the first place remains an active and unresolved problem in its own right: recent computational work shows that structural regularity, color variety, and semantic surprise each make separable, non-redundant contributions to perceived complexity, and that models relying on a single simple metric systematically fail to capture this multidimensionality [13]—a caution directly relevant to any study, including the present one, that operationalizes complexity through a single generative dimension. At the same time, structured regularity may support perceptual fluency, enabling more distributed exploration of visual scenes. The relationship between complexity and subjective evaluation has a long history in experimental aesthetics: Berlyne’s collative-variables framework proposed that stimulus properties such as complexity, novelty, and ambiguity determine an observer’s arousal potential, with hedonic value following an inverted-U function of arousal—stimuli of intermediate complexity being preferred over both very simple and very complex configurations [14]. This inverted-U prediction has, however, received markedly mixed empirical support in more recent work: a large-sample re-examination of complexity and liking in product design found scant evidence for the inverted-U pattern once cognitive effort, arousal, and perceived producer skill were accounted for, instead reporting a predominantly negative relationship between complexity and liking [15]—a direction more consistent with the pattern we report below than with Berlyne’s original formulation. More recent work within this tradition has emphasized processing fluency—the subjective ease with which a stimulus is perceptually or conceptually processed—as a proximal determinant of aesthetic liking, independent of the observer’s explicit awareness of the stimulus properties driving that ease [16]. A recent integrative review of the neuroaesthetics literature similarly frames aesthetic experience as emerging from the interaction of sensory-motor, emotion-valuation, and meaning-knowledge systems, each engaging distinct and only partially overlapping neural and behavioral signatures [17]—consistent with the possibility that evaluative judgment and overt attentional/oculomotor engagement, though often studied together, need not be driven by a single unified mechanism. These accounts converge on the prediction that structural manipulations of complexity should shape subjective aesthetic response, but they are comparatively agnostic about whether such manipulations also shape the overt allocation of gaze.

This last point is particularly relevant given a growing body of evidence that overt attention (where the eyes are directed) and covert evaluative or preferential processes can dissociate. In face perception research, for instance, covert and overt measures of attention to attractive faces have been shown to diverge depending on task demands [18], and developmental work on symmetry preference has found that although young children fixate symmetrical patterns longer than asymmetrical ones, they show no corresponding explicit preference for those patterns—a clear dissociation between spontaneous attentional capture and evaluative judgment [19]. These findings caution against the assumption that a structural property which reliably shapes subjective judgment will necessarily leave an equally reliable signature in fixation-based attentional metrics, or vice versa.

A complementary way of characterizing attentional deployment during free viewing is the distinction between ambient and focal modes of visual processing. Ambient viewing is characterized by short fixations followed by large-amplitude saccades and is thought to support the rapid extraction of a scene’s global spatial layout, whereas focal viewing involves longer fixations following short saccades and supports detailed, localized processing [20]. This distinction has been formalized in the coefficient *K*, a parametric index derived from the standardized relationship between fixation duration and the amplitude of the subsequent saccade, with positive values indicating focal and negative values indicating ambient processing [21]. Because harmony and complexity manipulations are natural candidates for shifting the balance between ambient and focal processing, the present study incorporates this framework alongside conventional eye-tracking metrics.

Despite these theoretical advances, relatively few studies have systematically isolated the effects of visual harmony and complexity using tightly controlled stimuli that eliminate semantic and contextual confounds. Many existing studies rely on real-world or artistic images, where multiple visual variables co-vary, making it difficult to attribute attentional effects to specific structural properties.

The present study addresses this gap by examining how controlled manipulations of visual structure modulate attentional allocation during free viewing. Using a set of stimuli derived from a common geometric base, we compare three conditions varying in harmony and complexity while maintaining constant low-level visual features. Eye-tracking measures—including time to first fixation (TTFF), fixation duration, number of fixations, scanpath entropy, and spatial dispersion—are used to characterize differences in visual exploration. To complement these overt gaze-based indices with an independent, non-oculomotor window onto attentional engagement, we additionally recorded webcam-based facial coding throughout stimulus presentation, yielding moment-by-moment estimates of neutral, happy, and surprise expressions together with the coefficient *K* described above.

We hypothesize that harmonious stimuli will elicit more distributed attentional patterns, whereas disorganized stimuli will induce focal attentional capture. A third condition of structured complexity is expected to produce intermediate patterns, reflecting a balance between global exploration and local processing. Given the dissociation reported between covert/overt attention and explicit preference in related domains [18,19], we also consider it plausible that subjective ratings of pleasantness and complexity may respond to the structural manipulation independently of, or even in the absence of, corresponding shifts in fixation-based attentional metrics. By isolating the role of visual structure and combining oculomotor, facial-coding, and subjective measures, this study aims to contribute to a more precise understanding of how formal properties guide attentional dynamics.

This study is intended to make two distinct kinds of contribution, and we flag both explicitly here so that the reader can evaluate each on its own terms. The first is substantive: as reported below, our attempt to manipulate complexity through structural disorder alone produced a pattern of subjective complexity judgments opposite to what was intended, suggesting that structural regularity/disorder is not interchangeable with perceived complexity as a construct—a dissociation with direct implications for how complexity is operationalized in eye-tracking and experimental-aesthetics research using generated or parametric stimuli. The second is methodological: analyzing this dataset surfaced two analytic issues—treating repeated within-participant observations as independent in correlational analyses, and the absence of an explicit multiplicity strategy across a large family of outcomes—that are common in webcam-based eye-tracking and facial-coding studies of this kind but are not always addressed. We report, in detail, how correcting for both changes the statistical picture (Section 3 and Section 4), with the aim of providing a worked example that other researchers analyzing similarly structured repeated-measures eye-tracking and facial-coding data can draw on directly.

## 2. Materials and Methods

### 2.1. Participants

A total of 50 participants were recruited through the RealEye online platform. All participants reported normal or corrected-to-normal vision and provided informed consent prior to participation. The study was conducted in accordance with the Declaration of Helsinki and approved by the corresponding institutional ethics committee.

Given the use of webcam-based eye tracking, participants were required to complete a calibration procedure prior to the experiment. Data quality was evaluated using RealEye’s built-in quality grade (a 1–6 composite index of calibration accuracy and data completeness). Only participants with a quality grade ≥4 were retained for analysis, resulting in a final sample of N=40 (age range: 19–32 years, M=22.11, SD=2.92; 25 female, 14 male, 1 other), with the remaining 10 participants excluded.

### 2.2. Stimuli

The stimulus set consisted of nine images (three exemplars per condition) derived from a common geometric base and generated through a parametric tessellation procedure, systematically manipulated to vary in visual structure while controlling for low-level features. All stimuli were rendered at the same resolution and aspect ratio and were matched in overall luminance, color palette (a shared orange–teal–cream scheme), and approximate number of visual elements, so that the three conditions differed primarily in their spatial and structural organization rather than in low-level photometric properties.

**Figure 1:**
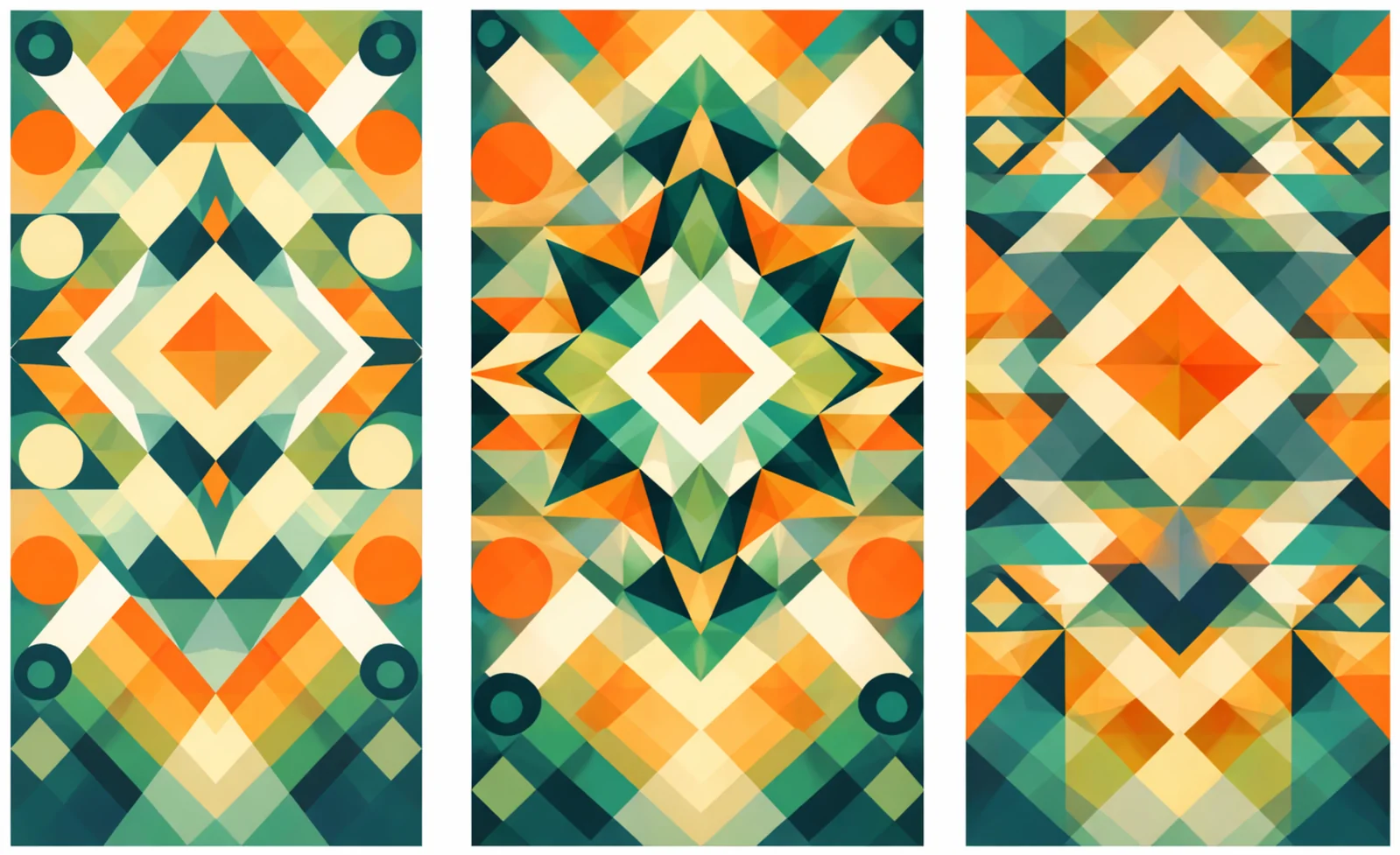
Examples of geometric patterns with high harmony.

**Figure 2:**
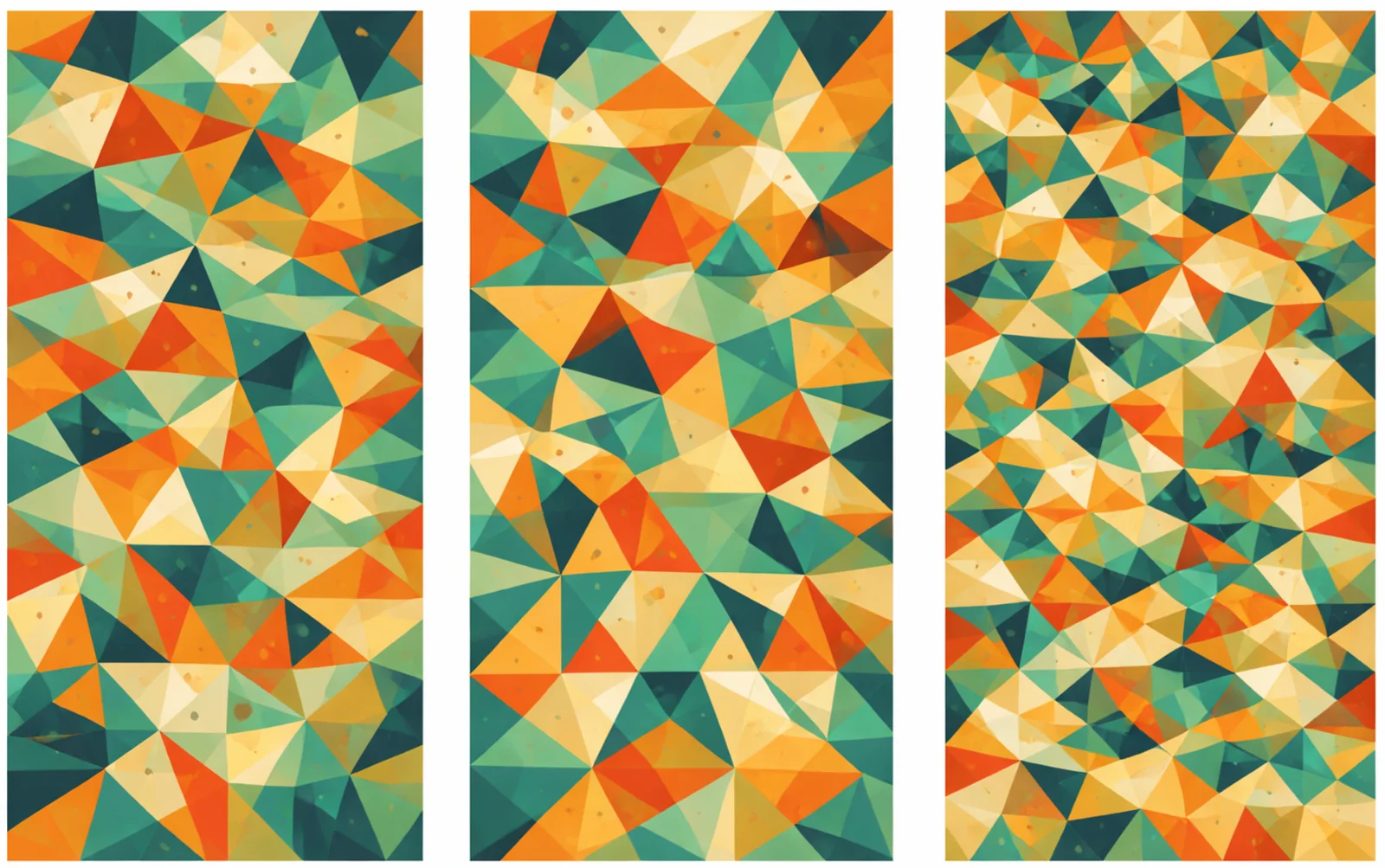
Examples of geometric patterns with high complexity and low harmony.

**Figure 3:**
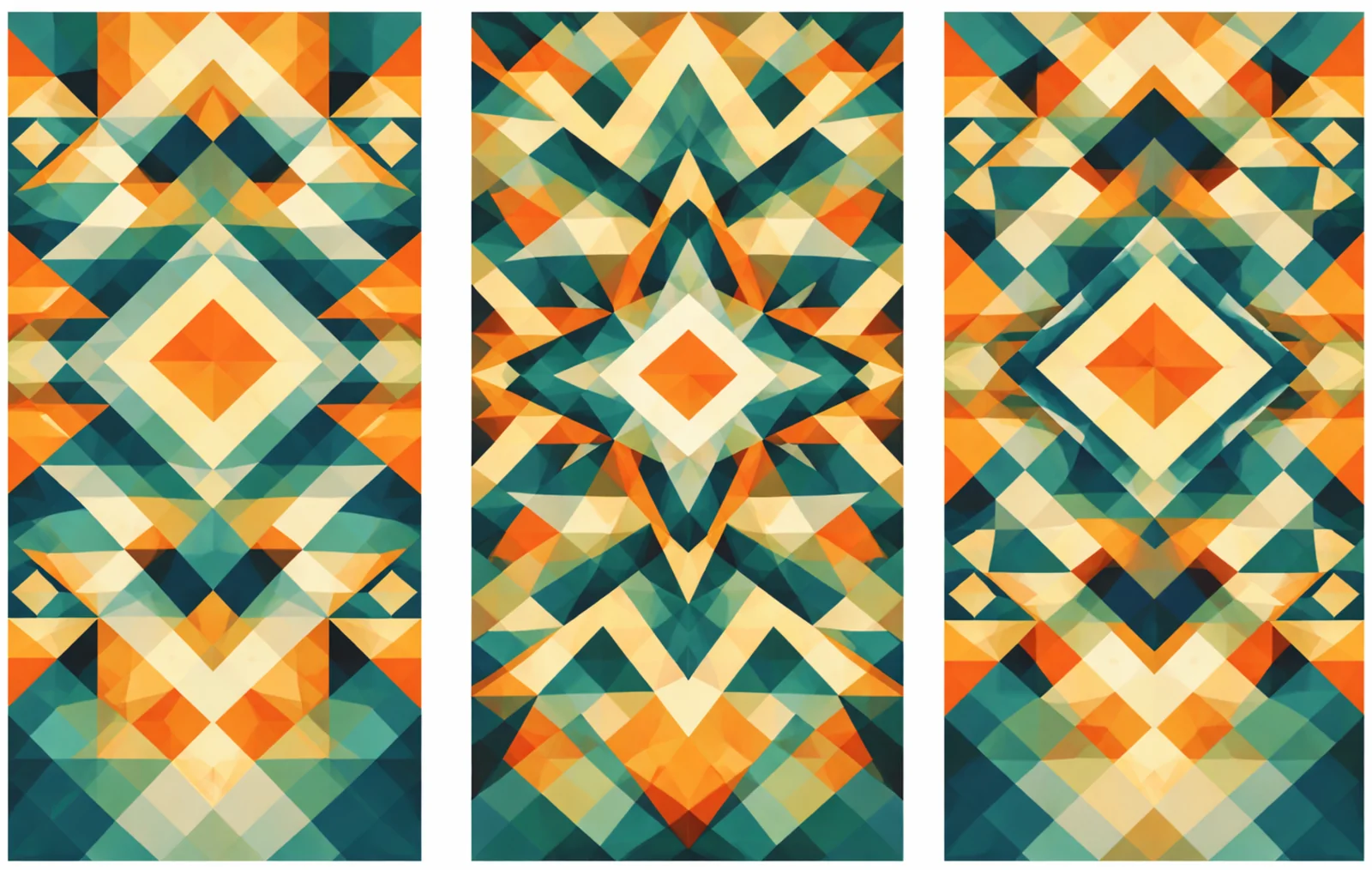
Examples of geometric patterns with structured complexity.

The three experimental conditions were:

- High harmony (low complexity, HH): A symmetrical and highly organized composition built around a central radial motif, characterized by regular spatial distribution, mirror symmetry along both the vertical and horizontal axes, and smooth visual transitions between color regions (see Figure 1).
- High complexity (low harmony, HC): A disorganized tessellation of small, irregularly sized triangular facets covering the same canvas, with disrupted symmetry, non-uniform spatial arrangement, and increased local contrast between adjacent facets, yielding a texture-like appearance without a clear global focal point (see Figure 2).
- Structured complexity (SC): A condition combining a high density of small geometric facets, similar in scale to the HC condition, with an underlying global symmetry and a salient central motif analogous to that of the HH condition—that is, local complexity embedded within global order (see Figure 3).

The visual similarity between HH and SC relative to HC is a deliberate feature of this design rather than an oversight. SC was constructed specifically to test whether local complexity could be embedded within global order without disrupting harmony-related structure; to isolate that manipulation, SC retains the same global mirror symmetry and central radial motif as HH, differing from it primarily in the density and irregularity of the small facets that compose that structure. HC, by contrast, removes the global symmetry and central motif entirely. Harmony and complexity were therefore defined a priori in purely generative, geometric terms—degree of mirror symmetry, presence/absence of a central motif, and the size and irregularity of the constituent facets—rather than through a preliminary psychophysical scaling of participants’ own perception of these dimensions. This is an important distinction: as reported in Section 3.2 and discussed in Section 4, this a priori structural classification did not fully align with participants’ subjective experience of complexity, indicating that the correspondence between generative parameters and perceived harmony/complexity cannot simply be assumed. We return to this point, and to how it should be addressed in future stimulus development (e.g., via an independent pilot rating study prior to the main experiment), in Section 4.

All stimuli were presented in full-screen format and standardized to identical viewing conditions. No areas of interest (AOIs) were predefined on the stimuli; all eye-tracking metrics were therefore computed over the entire stimulus area rather than for specific sub-regions (see Section 2.5).

### 2.3. Apparatus

Eye movements were recorded using the RealEye platform, a webcam-based eye-tracking system with an approximate sampling rate of 30 Hz. Although less precise than laboratory-grade systems, RealEye has been shown to provide reliable measures of fixation patterns and attentional distribution in remote experimental settings. Fixations were identified with RealEye’s default filter settings (gaze-point interpolation at 30 Hz with a maximum gap of 50 ms, a velocity threshold of 150°/s, and minimum and maximum fixation durations of 80 and 400 ms, respectively). We retained these platform defaults, rather than a custom filter, to keep fixation identification consistent with the calibration and validation benchmarks reported for RealEye and to avoid introducing additional researcher degrees of freedom into a webcam-based signal that is already noisier than laboratory-grade recordings. The upper bound of 400 ms is within the range commonly used for dispersion-based fixation classifiers, but it will truncate or split any genuine fixations that exceed it; because sustained fixations beyond 400 ms are, if anything, more likely during effortful or difficult processing, this truncation could attenuate rather than inflate condition differences in mean fixation duration, and we return to this as a limitation on our fixation-duration measure in Section 4.

The hardware and software constraints of this webcam-based approach do affect spatial and temporal precision, and this is worth making explicit. RealEye’s own validation reports a full-screen spatial accuracy on the order of 100–125 px (corresponding to roughly 2–5 degrees of visual angle depending on viewing distance and screen size, both uncontrolled in this remote setting), compared with sub-degree accuracy typical of laboratory-grade infrared trackers, and a sampling rate (here, ∼30 Hz) an order of magnitude lower than the 250–1000 Hz common in laboratory systems. Consistent with this, a direct comparison of webcam-based (including RealEye) and remote infrared eye tracking reported higher measurement error and reduced precision for the webcam modality, although effects large enough to be behaviorally meaningful (e.g., differences between highly discriminable stimulus categories) were still reliably detected under both modalities [22]. Similarly, a direct comparison against a laboratory EyeLink 1000 system found that a modern webcam-based tracker approaches, without fully matching, laboratory-grade spatial accuracy [23], and independent behavioral replications of classic in-lab eye-tracking paradigms using webcam-based, browser-delivered tracking have reported little degradation in the size of well-established effects despite reduced spatial and temporal resolution [24]. This lower spatial/temporal precision and correspondingly reduced signal-to-noise ratio is a plausible contributor to the null condition effects on eye-tracking metrics reported in Section 3.1, and we return to it explicitly in Section 4.

In parallel with eye tracking, RealEye’s facial-coding module recorded participants’ facial expressions from the webcam feed throughout each trial, sampled at the same 30 Hz rate. Facial coding was based on the Facial Action Coding System (FACS) [25] and yielded, for every 30-ms sample, the estimated intensity (0–1) of three expression categories, neutral, happy, and surprise, together with an instantaneous, standardized estimate of the coefficient *K* [21], an index of ambient (negative values) versus focal (positive values) visual-attentional engagement derived jointly from oculomotor dynamics.

### 2.4. Procedure

Participants completed the experiment remotely using a desktop or laptop computer equipped with a webcam. After providing consent, they performed a standard nine-point calibration procedure to ensure eye-tracking accuracy; webcam access was also required and calibrated for facial coding.

Each trial consisted of the presentation of a single stimulus for a duration of 6–7 s under free-viewing conditions. This duration was chosen to be long enough to allow multiple fixations and at least one full exploratory pass over each stimulus (yielding, in practice, a mean of approximately 24 fixations per trial; see Section 3.1) while remaining short enough, across nine trials plus ratings, to limit fatigue in a webcam-based remote setting where sustained attention and calibration quality tend to degrade over longer sessions. Whether 6–7 s is sufficient for global structural properties (as opposed to local, early-fixation salience) to shape the overall distribution of fixations is, however, an open question that this design cannot fully resolve; we consider this explicitly as a candidate explanation for the null eye-tracking results in Section 4. The nine stimuli (three exemplars × three conditions) were presented once each, in an order randomized independently for every participant to control for order and fatigue effects.

Following each stimulus, participants completed a brief set of subjective ratings:

- Perceived pleasantness (1–5 Likert scale)
- Perceived complexity (1–5 Likert scale)
- (Optional) First perceived element (categorical response)

No specific instructions were given regarding where to look, in order to capture spontaneous attentional behavior. Participants were informed, as part of the consent process and prior to the experiment, that the platform would analyze their facial expressions from the webcam feed in addition to tracking their gaze; consistent with RealEye’s privacy-preserving design, no video was recorded or stored at any point—the platform processes the live webcam feed on the client side and returns only derived gaze coordinates and facial-expression estimates. The ethics approval covering this study explicitly included this webcam-based facial-expression analysis alongside eye tracking.

### 2.5. Measures

The primary dependent variables were derived from eye-tracking data. Because no areas of interest were predefined on the stimuli (Section 2.2), all metrics below were computed over fixations landing anywhere within the presentation area:

- Time to First Fixation (TTFF): latency, from stimulus onset, to the first recorded fixation.
- Fixation Duration: mean duration of fixations per stimulus.
- Number of Fixations: total number of fixations during stimulus presentation.
- Scanpath Entropy: Shannon entropy of the spatial distribution of fixations across a 5×5 grid superimposed on the stimulus, normalized by its theoretical maximum ($\log_{2}25$); higher values indicate more spatially distributed viewing.
- Heatmap Dispersion: spatial dispersion of fixation coordinates, computed as $\sqrt{\mathrm{Var}(x)+\mathrm{Var}(y)}$, where *x* and *y* are fixation coordinates expressed as a percentage of stimulus width and height.

From the facial-coding data, the following measures were derived for each participant and stimulus: the mean intensity of neutral, happy, and surprise expressions across the trial, and the trial-level mean of the coefficient *K* (RealEye’s aggregated per-trial estimate).

Additionally, subjective ratings of perceived pleasantness and complexity were recorded for each stimulus. For all measures, condition-level scores were computed by averaging across the three exemplars of each condition, separately for every participant.

### 2.6. Data Analysis

Statistical analyses were conducted using a repeated-measures design. Differences across the three stimulus conditions were evaluated using one-way repeated-measures ANOVA for each dependent variable (eye-tracking metrics, facial-coding metrics, and subjective ratings), with condition-level scores obtained by averaging across the three exemplars per condition for each participant.

Post-hoc comparisons were performed using paired-samples *t*-tests with Bonferroni correction for the three pairwise comparisons per variable. Effect sizes were reported using partial eta squared ($\eta_{p}^{2}$) for omnibus tests and Cohen’s *d* for pairwise comparisons.

Because averaging over exemplars conflates condition with the specific stimulus items used to instantiate it, and because only three exemplars were sampled per condition, we additionally fit linear mixed-effects models with crossed random intercepts for participant and item (i.e., not averaging across exemplars) for each primary dependent variable, with condition modeled as a fixed effect (treatment-coded contrasts against the HH baseline). Omnibus condition effects were evaluated via likelihood-ratio tests comparing models with and without the condition fixed effect, both fit by maximum likelihood; parameter estimates are reported from the corresponding restricted maximum likelihood (REML) fits. This analysis serves as a robustness check on the repeated-measures ANOVA results by explicitly partitioning item-level variance rather than treating it as part of the residual.

To contextualize the null eye-tracking results, we conducted a sensitivity power analysis based on the present design (N=40, $df_{1}=2$, $df_{2}=78$, α=0.05), determining the minimum partial eta squared for which the study had 80% power, using the noncentral *F* distribution. We emphasize that this analysis was necessarily conducted post hoc, after the null pattern was observed, rather than as a prospective sample-size justification prior to data collection; no such prospective power calculation was performed when the study was designed. Consequently, the sensitivity analysis should be read only as evidence of what the present design could and could not detect, not as a substitute for a pre-registered power analysis, and the absence of significant eye-tracking or facial-coding effects is interpreted throughout as an absence of detectable differences under the present design and sample size, rather than as evidence that visual structure has no effect on attention or facial engagement [26].

Correlational analyses examined relationships between subjective ratings (pleasantness and complexity), eye-tracking metrics, and facial-coding metrics. Because each of the 40 retained participants contributed one observation per condition (i.e., three non-independent observations per participant, N=120 condition-level observations in total), treating these as 120 independent data points—as an ordinary Pearson correlation implicitly does—violates the independence assumption underlying that test and can bias both the point estimate and its associated *p*-value. We therefore used repeated-measures correlation (rmcorr) as our primary correlational technique [27], which estimates the common within-participant association between two variables while explicitly partitioning out between-participant variance, analogous to an ANCOVA with participant modeled as a categorical factor. Because we lacked access to the original rmcorr software (https://lmarusich.github.io/rmcorr/ (accessed on 8 September 2026)) in the offline analysis environment used here, we implemented the equivalent ANCOVA-based procedure directly (participant fixed intercepts plus a common slope, fit by ordinary least squares; the partial correlation $r_{rm}$ and its associated *F*-test were derived from the reduction in residual sum of squares attributable to the slope term, with $df_{2}=N-k-1$ for k=40 participants). For comparison, we also report the naive Pearson correlation obtained by treating the 120 observations as independent, to make transparent how much the correction changes each estimate.

Given the number of statistical tests performed across outcome families, we distinguish primary from exploratory analyses and adjust for multiplicity accordingly. This designation of primary versus exploratory outcomes reflects which dependent variables the Introduction’s hypotheses target, rather than a formally pre-registered analysis plan. The seven primary tests correspond to the central hypotheses stated in the Introduction and are the repeated-measures ANOVAs on the five eye-tracking metrics and the two subjective ratings; for this family we report both the nominal *p*-value and significance under a Bonferroni-corrected threshold (α=0.05/7=0.00714). The four facial-coding ANOVAs are treated as a secondary, exploratory family, since facial coding was introduced to complement the primary oculomotor and subjective measures rather than to test an independently specified hypothesis. All correlational analyses (23 rmcorr tests across eye-tracking, facial-coding, and subjective variables) are likewise treated as exploratory and are corrected using the Benjamini–Hochberg false discovery rate (FDR) procedure across that full set of 23 tests; we report FDR-adjusted *q*-values alongside nominal *p*-values and flag which associations survive correction. Exploratory findings, whether or not nominally significant, are interpreted as hypothesis-generating rather than confirmatory.

Significance level was set at α=0.05 for all analyses. All analyses were performed in Python v3.14.7 (NumPy, SciPy, and pandas); linear mixed-effects models and the repeated-measures correlation procedure were implemented directly via profile REML/ML and ordinary-least-squares optimization, respectively, since packages providing this functionality (e.g., statsmodels, lme4, rmcorr) were not available in the analysis environment.

## 3. Results

### 3.1. Eye-Tracking Measures

**Figure 4:**
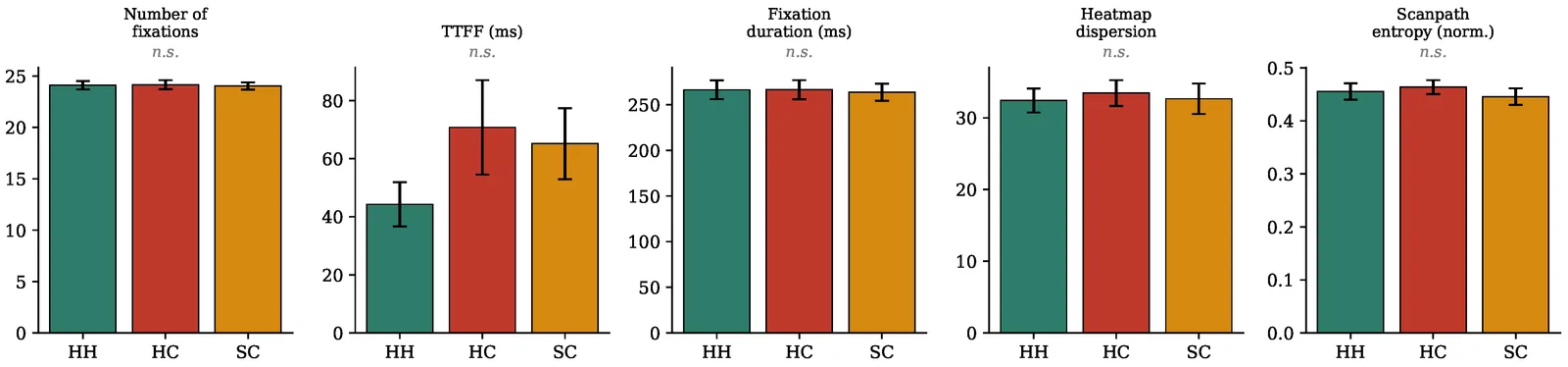
Eye-tracking metrics by condition (mean ± SEM, N=40). None of the five metrics differed significantly across conditions (repeated-measures ANOVA, all p>0.15; n.s. = not significant).

**Figure 5:**
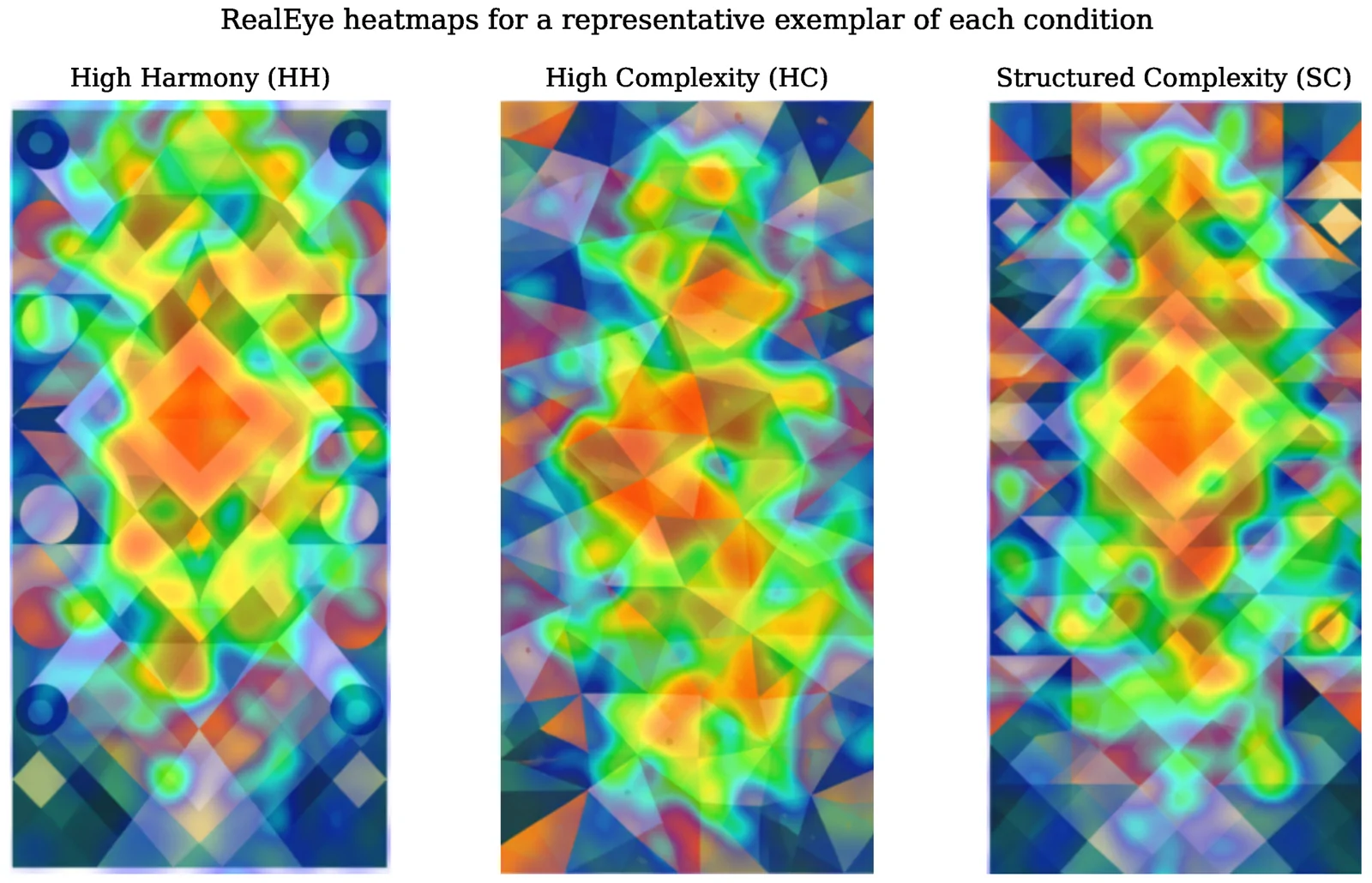
RealEye-generated heatmaps overlaid on a single representative exemplar (Image 1) of each condition. These platform-generated heatmaps are shown for illustrative purposes only; the statistical analyses reported below are based on the full aggregated dataset (all three exemplars, N=40 participants per condition), summarized quantitatively in Figure 6.

**Figure 6:**
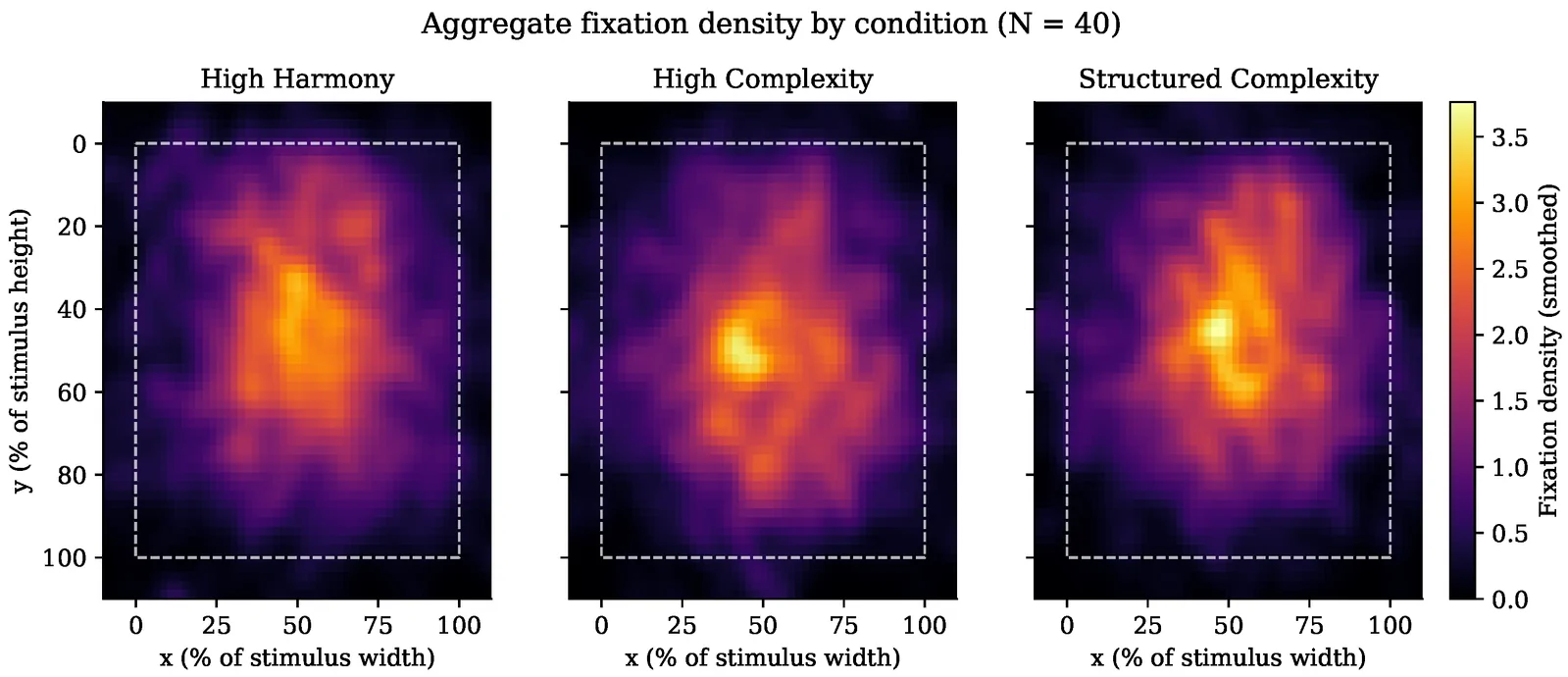
Aggregate fixation density (smoothed 2D histograms) across all 40 participants, by stimulus condition. Dashed rectangles indicate the stimulus boundaries. Color scale is shared across panels to allow direct visual comparison of spatial spread.

A series of one-way repeated-measures ANOVAs compared the three stimulus conditions (high harmony, HH; high complexity, HC; structured complexity, SC) on each eye-tracking metric, averaged across the three exemplars per condition. Contrary to our hypotheses, none of the five eye-tracking metrics showed a statistically significant effect of condition (Table 1, Figure 4). Descriptively, time to first fixation was numerically shortest for HH (M=44.3 ms) relative to HC (M=70.8 ms) and SC (M=65.2 ms), but this difference did not reach significance, and no pairwise comparison survived Bonferroni correction for any metric. Single-exemplar heatmaps such as those in Figure 5 can give the visual impression of a more fragmented, multi-peaked distribution for HC relative to the single dominant hotspot in HH and SC; however, this impression did not translate into a statistically reliable difference once fixations were aggregated across all three exemplars and all 40 participants (Figure 6), underscoring the importance of quantitative aggregation over qualitative inspection of individual stimuli. Inspection of the remaining exemplars reinforces this point: whereas all three HH exemplars showed a consistent single-hotspot pattern and all three HC exemplars showed a consistent fragmented, multi-peaked pattern, the SC exemplars were less consistent with one another—two resembled the single-hotspot pattern typical of HH, while the third more closely resembled the fragmented pattern typical of HC—plausibly reflecting the condition’s intended combination of local complexity with global structure, and consistent with SC’s descriptive values falling consistently between HH and HC across the quantitative metrics in Table 1. All three conditions in the aggregated maps show a similarly concentrated, centrally biased distribution of fixations, without the more distributed pattern predicted for HH or the more focal pattern predicted for HC.

Are these uniformly null effects reasonable given the experimental setting, rather than symptomatic of a failed measurement? We believe so, for two convergent reasons developed fully in Section 4: (i) the sensitivity analysis in Section 3.4 shows the design was powered to detect only medium-to-large effects ($\eta_{p}^{2}\geq 0.114$), well above the small effects actually observed ($\eta_{p}^{2}=0.003$–0.046); and (ii) the webcam-based sampling rate and spatial accuracy characterized in Section 2.3 are appreciably coarser than laboratory-grade systems, which would attenuate—though not necessarily eliminate—small true condition differences, a limitation independently documented for this class of platform [22]. We note that an earlier version of this argument additionally cited the coefficient *K*’s correlation with independent oculomotor indices as evidence that the measurement pipeline was sensitive to genuine oculomotor structure; as detailed in Section 3.6, that correlation does not survive correction for the non-independence of repeated observations, and we no longer rely on it here. Together, the two reasons above suggest the null pattern reflects a combination of limited statistical power and instrument precision rather than a wholesale failure of the eye-tracking or facial-coding measures.

### 3.2. Subjective Ratings

**Figure 7:**
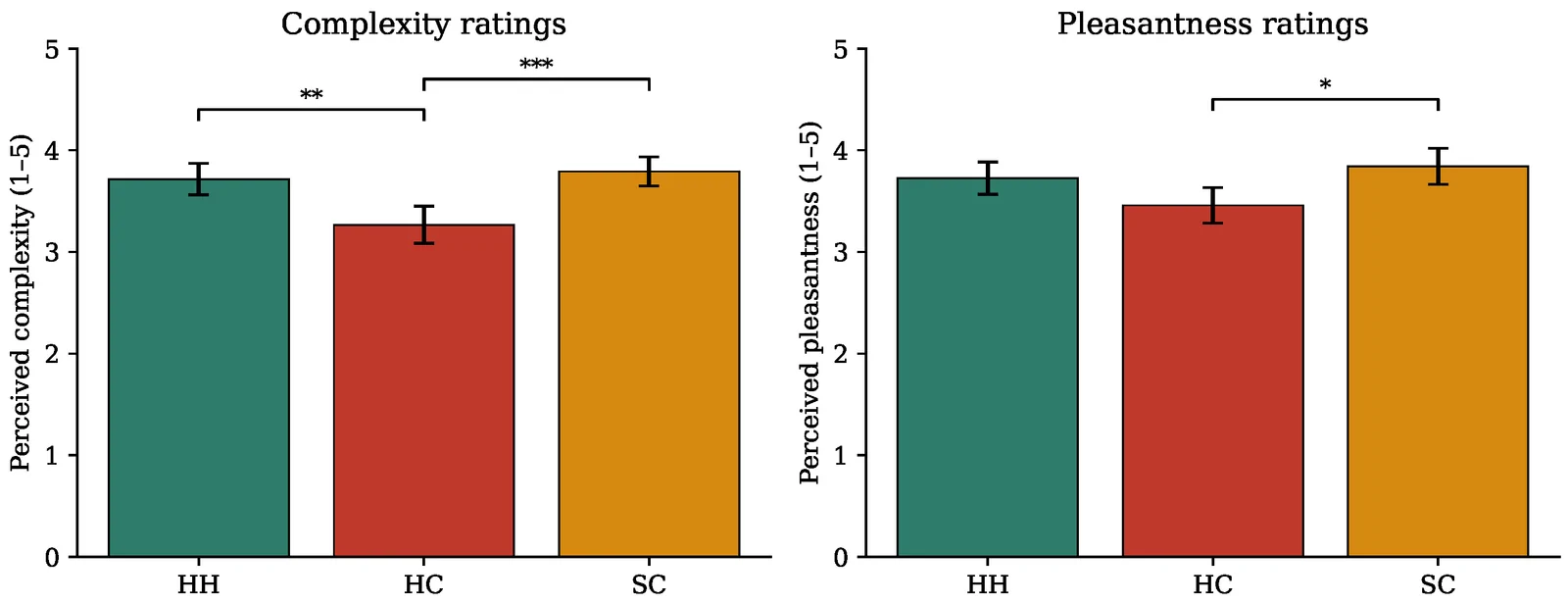
Subjective ratings by condition (mean ± SEM, N=40). Brackets show Bonferroni-corrected pairwise comparisons (* p<0.05, ** p<0.01, *** p<0.001).

Perceived complexity differed significantly across conditions, F(2,78)=11.10, p<0.001, $\eta_{p}^{2}=0.22$ (Figure 7). Bonferroni-corrected pairwise comparisons showed that HC was rated as less complex (M=3.27, SD=1.15) than both HH (M=3.72, SD=0.97; t=3.33, $p_{bonf}=0.006$, d=0.53) and SC (M=3.79, SD=0.91; t=−3.84, $p_{bonf}=0.001$, d=−0.61), which did not differ significantly from each other ($p_{bonf}=1.00$). This pattern is opposite to the intended manipulation, in which HC was designed to be the most complex condition. This effect survives the stringent Bonferroni-corrected threshold for the family of seven primary omnibus tests described in Section 2 (α=0.00714), and is accordingly treated as the most robust finding of the study.

Perceived pleasantness also differed significantly across conditions, F(2,78)=3.71, p=0.029, $\eta_{p}^{2}=0.087$, driven primarily by SC being rated as more pleasant (M=3.84, SD=1.12) than HC (M=3.46, SD=1.11; t=−2.52, $p_{bonf}=0.048$, d=−0.40). HH (M=3.73, SD=1.01) did not differ significantly from either HC or SC after correction. Unlike the complexity effect, this omnibus result does *not* survive the Bonferroni-corrected threshold for the seven-test primary family (p=0.029>0.00714); we therefore treat the pleasantness effect as a nominally significant but comparatively fragile finding, and interpret it with corresponding caution throughout the remainder of this article.

### 3.3. Relationship Between Subjective Ratings and Eye-Tracking Metrics

Because each participant contributed one observation per condition, the 120 condition-level observations analyzed here are not independent, and naive Pearson correlations computed directly on them can be misleading (Section 2). We therefore report repeated-measures correlations ($r_{rm}$) as the primary statistic, alongside the naive Pearson *r* for reference, and treat this entire set of 23 correlational tests (Section 3.3, Section 3.4, Section 3.5 and Section 3.6) as exploratory, applying Benjamini–Hochberg FDR correction across the full set (Section 2).

**Figure 8:**
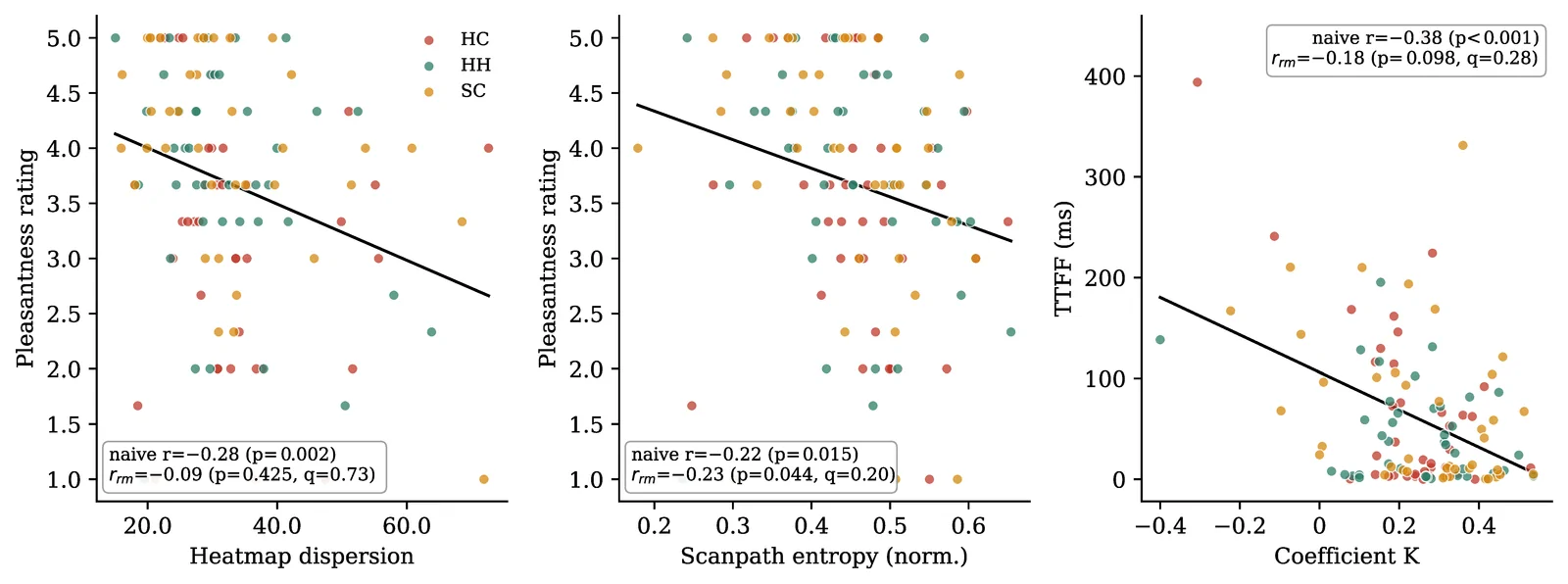
Key correlations at the condition level (each point is one participant × condition observation, N=120, from k=40 participants). Naive Pearson *r* treats all 120 observations as independent; $r_{rm}$ is the repeated-measures correlation that accounts for the fact that each participant contributed three non-independent observations, with its associated *p*-value and the Benjamini–Hochberg FDR-adjusted *q*-value across the full 23-test exploratory correlation family (Section 2). None of the three associations shown survives FDR correction (q>0.05 throughout).

Under the naive Pearson analysis, heatmap dispersion (r=−0.28, p=0.002) and scanpath entropy (r=−0.22, p=0.015) both appeared negatively correlated with pleasantness, contrary to the exploratory-processing account proposed in the Introduction. Once the repeated-measures structure is accounted for, this pattern weakens substantially (Figure 8): the dispersion–pleasantness association is no longer distinguishable from zero ($r_{rm}=-0.09$, F(1,79)=0.64, p=0.425, q=0.735), while the entropy–pleasantness association remains directionally similar and nominally significant ($r_{rm}=-0.23$, F(1,79)=4.20, p=0.044) but does not survive FDR correction across the 23-test exploratory family (q=0.201). No eye-tracking metric showed a robust association with perceived complexity: fixation duration was nominally associated with complexity ($r_{rm}=0.24$, p=0.029) but this likewise does not survive FDR correction (q=0.193), and all remaining associations (number of fixations, TTFF, heatmap dispersion, and scanpath entropy with complexity) had p>0.06 under rmcorr. We therefore treat the entire pattern of eye-tracking–rating correlations as, at most, weak and exploratory evidence rather than a confirmed dissociation, and revisit the interpretive implications of this correction in Section 4.

### 3.4. Robustness: Item-Level Mixed-Effects Models and Power Sensitivity

Because the repeated-measures ANOVAs above averaged across the three exemplars per condition, we re-analyzed each primary eye-tracking and subjective-rating variable at the level of individual participant–item observations (N=40 participants × 9 items), using linear mixed-effects models with crossed random intercepts for participant and item and condition as a fixed effect. This addresses the possibility that averaging over only three exemplars per condition conflates condition with idiosyncratic item effects. The pattern of results was consistent with the condition-level ANOVAs (Table 2): none of the five eye-tracking metrics showed a significant omnibus effect of condition (all likelihood-ratio p>0.15), whereas both pleasantness ($\chi^{2}\left(2\right)=12.05$, p=0.002) and complexity ($\chi^{2}\left(2\right)=8.14$, p=0.017) ratings did, with the same direction of effects reported above (HC lower than HH and SC on both measures). Estimated item-level variance components were negligible relative to participant-level variance for all eye-tracking metrics, indicating that the specific exemplars used did not introduce meaningful additional variability beyond that already captured by condition and participant.

Given the null eye-tracking results, we conducted a sensitivity power analysis to establish what magnitude of effect the present design was actually equipped to detect. With N=40 and the present repeated-measures structure ($df_{1}=2$, $df_{2}=78$), the study had 80% power at α=0.05 to detect effects of $\eta_{p}^{2}\geq 0.114$ (a medium-to-large effect by conventional benchmarks). All five eye-tracking metrics yielded observed effect sizes well below this threshold ($\eta_{p}^{2}=0.003$–0.046), indicating that the present sample size was not equipped to reliably detect small effects in the range actually observed, and that a true small effect of visual structure on overt attentional allocation cannot be ruled out. By contrast, the effect sizes for pleasantness ($\eta_{p}^{2}=0.087$) and, especially, complexity ratings ($\eta_{p}^{2}=0.222$) approached or clearly exceeded this threshold, which is consistent with why these effects were reliably detected while the eye-tracking effects were not.

### 3.5. Facial-Coding Measures

To complement the oculomotor metrics with an independent index of attentional and affective engagement, facial-coding data (neutral, happy, and surprise expression intensity, and the coefficient *K*) were analyzed for the same 40 participants (Table 3). As with the eye-tracking metrics, none of the four facial-coding measures differentiated the three structural conditions: neutral, F(2,78)=1.12, p=0.333, $\eta_{p}^{2}=0.028$; happy, F(2,78)=0.89, p=0.415, $\eta_{p}^{2}=0.022$; surprise, F(2,78)=0.72, p=0.490, $\eta_{p}^{2}=0.018$; coefficient *K*, F(2,78)=0.40, p=0.670, $\eta_{p}^{2}=0.010$. No pairwise comparison survived Bonferroni correction for any measure.

As in Section 3.3, correlations between facial-coding measures and subjective ratings are reported here as repeated-measures correlations ($r_{rm}$), with naive Pearson *r* given for reference, and evaluated against the Benjamini–Hochberg FDR threshold across the full 23-test exploratory correlation family (Section 2). Although facial-coding measures showed no condition effect, none correlated robustly with either subjective rating. With pleasantness: happy (naive r=−0.01; $r_{rm}=-0.08$, p=0.482), surprise (naive r=0.06; $r_{rm}=-0.02$, p=0.888), neutral (naive r=0.13; $r_{rm}=-0.02$, p=0.834), and *K* (naive r=−0.11; $r_{rm}=-0.07$, p=0.512). With complexity: happy ($r_{rm}=0.08$, p=0.504), neutral ($r_{rm}=0.20$, p=0.067), and *K* ($r_{rm}=0.12$, p=0.272) were all non-significant; surprise showed a nominally significant association ($r_{rm}=0.24$, p=0.032) that did not survive FDR correction (q=0.193).

These null facial-coding correlations, like the null condition effects reported above, are, we think, readily explicable rather than anomalous. First, the absolute expression intensities were uniformly low and close to floor for happy (0.08–0.10) and surprise (0.02) across all three conditions (Table 3); validation studies of FACS-based and FACS-derived automated coding consistently show it performs best on posed or spontaneous expressions of the basic emotions in social or emotionally evocative contexts [25,28], and a documented limitation of automated facial-expression analysis more broadly is its reliance on an assumption of coherence between a discrete emotional state and a corresponding facial display [29]—an assumption that may simply not hold for observers viewing abstract, non-representational geometric patterns, which give little reason to produce any overt facial expression in the first place, regardless of structural condition. With so little expressive variance to work with, detecting a subtle condition effect—even a genuine one—becomes correspondingly less likely. Second, the same power limitation that applies to the eye-tracking metrics (Section 3.4) applies here: the facial-coding effect sizes ($\eta_{p}^{2}=0.01$–0.03) fall even further below the $\eta_{p}^{2}\geq 0.114$ threshold for which this design had adequate power than the eye-tracking effects did. Third, the coefficient *K* derived from this same facial-coding pipeline shows a mixed relationship with independent oculomotor indices once the repeated-measures structure is accounted for (Section 3.6); we discuss the implications of this more cautiously than in earlier analyses.

### 3.6. Convergence Between the Coefficient *K* and Oculomotor Indices

As above, we report repeated-measures correlations ($r_{rm}$) as the primary statistic, with naive Pearson *r* for reference and FDR-adjusted *q*-values from the full 23-test exploratory family (Section 2). Although the coefficient *K* did not distinguish the three structural conditions, the naive (uncorrected) Pearson correlations suggested it was associated with several oculomotor metrics in the direction predicted by the ambient/focal framework [20,21]: shorter TTFF (naive r=−0.38, p<0.001), longer fixation durations (naive r=0.27, p=0.003), and greater heatmap dispersion (naive r=0.21, p=0.021). Once the non-independence of the 120 condition-level observations is accounted for via repeated-measures correlation, this pattern changes substantially (Table 4). The association with TTFF weakens to a non-significant trend ($r_{rm}=-0.19$, p=0.098, q=0.281); the association with heatmap dispersion is effectively eliminated ($r_{rm}=0.02$, p=0.879, q=0.961); and, most notably, the association with fixation duration not only loses significance under the naive interpretation but reverses sign ($r_{rm}=-0.24$, p=0.034), the opposite of what the ambient/focal framework predicts, though this reversed association likewise does not survive FDR correction (q=0.193). A previously non-significant association with number of fixations instead emerges as the numerically strongest repeated-measures correlation in this set ($r_{rm}=0.30$, p=0.007), though it too does not survive FDR correction (q=0.170). The correlation with scanpath entropy remained non-significant under both approaches (naive r=0.12, p=0.19; $r_{rm}=-0.02$, p=0.834).

Taken together, once the appropriate repeated-measures correlation and multiple-comparison correction are applied, none of the associations between coefficient *K* and the independent oculomotor indices reaches statistical significance, and the one metric for which a naive association had appeared strongest and most theoretically consistent (fixation duration) instead shows a nominally significant association in the opposite direction. We had previously interpreted the naive pattern as evidence of construct validity for the combined webcam-based measurement approach; in light of this correction, we no longer consider that interpretation well supported, and withdraw it as a load-bearing argument in the remainder of this article (see Section 4 for how this changes our interpretation of the null condition effects).

### 3.7. Descriptive Statistics for Individual Stimulus Exemplars

All statistics reported above are based on condition-level scores obtained by averaging across the three exemplars of each condition (Section 2.5) or, for the mixed-effects models in Section 3.4, on the full set of participant–item observations without such averaging. For transparency, Table 5 reports descriptive statistics (mean and SD across the N=40 retained participants) for each of the five eye-tracking metrics and the two subjective ratings, broken down by the nine individual stimulus exemplars rather than aggregated by condition. Consistent with the negligible item-level variance components reported in Section 3.4, the exemplar-level means within each condition are broadly similar to one another for the eye-tracking metrics, with the exception of a numerically longer TTFF for Image 1 of the HC condition (M=102.3 ms) relative to its two counterparts (M=51.5 and 58.6 ms); this single exemplar also contributed to the visual impression of a fragmented fixation pattern discussed in Section 3.1. For the subjective ratings, exemplar-level complexity scores are lowest for all three HC images relative to their HH and SC counterparts, indicating that the reversed complexity manipulation reported in Section 3.2 is not driven by a single outlying exemplar but is consistent across all three HC images.

## 4. Discussion

This study examined whether controlled variations in visual harmony and complexity, isolated from semantic content, would relate to overt attentional allocation during free viewing, and complemented conventional eye-tracking metrics with webcam-based facial coding and the ambient/focal coefficient *K*. Of the seven primary, family-wise-corrected omnibus tests (Section 2), only one—perceived complexity—survived correction, in a direction opposite to the intended manipulation; the pleasantness effect and the correlational associations discussed below are, at most, nominally significant and did not survive appropriate correction for multiple comparisons or for the non-independence of repeated observations (Section 2). None of the eye-tracking or facial-coding metrics differentiated the three structural conditions at even the nominal level. This pattern is best summarized not as a confirmed dissociation between overt attentional/affective signals and evaluative judgment—the framing our uncorrected initial analyses suggested—but as a single robust effect on subjective complexity judgment, several weaker and largely non-robust signals elsewhere, and no detectable condition effect on overt attention or facial engagement under the present design. This calls for a substantial revision of the framework proposed in the Introduction, and, as elaborated below, is at least broadly consistent with prior work showing that attention and preference need not move together [18,19], though our own findings support that claim far more tentatively than we originally reported.

### 4.1. The Complexity Manipulation Did Not Translate into Perceived Complexity

The most striking result is that the high-complexity (HC) condition was rated as less complex than both the high-harmony (HH) and structured-complexity (SC) conditions—the opposite of the intended manipulation. Several non-mutually-exclusive explanations are possible. First, the HC stimuli, generated as disordered tessellations of small triangular elements, may have produced a visually homogeneous texture in which the repetition of similarly sized units was perceived as more uniform (and therefore less complex) than the layered, multi-scale symmetry of the HH and SC stimuli, which combine large and small elements and salient central motifs. This would be consistent with classic findings that fixation counts and durations scale with structural complexity as defined by the number of distinguishable sides or elements in a shape, rather than with disorder per se [5], and with evidence that the perceived “goodness” of a pattern is closely tied to its degree of symmetry independent of its element count [4]. This interpretation is also consistent with recent computational work showing that structural regularity, color variety, and semantic surprise are separable, non-redundant contributors to perceived complexity [13]: our generative manipulation targeted structural disorder specifically, but disorder alone is evidently not synonymous with the complexity that participants reported perceiving. Irregular tessellation may increase disorder while paradoxically reducing the number of subjectively distinguishable units, thereby lowering rather than raising perceived complexity. Second, if symmetry detection itself depends on attentional resources rather than occurring preattentively [8], the salient bilateral symmetry of the HH and SC stimuli may have drawn attention to their internal structure in a way that made their local detail more, not less, apparent to observers—again working against the intended complexity ordering. Third, without a pilot manipulation check on this specific stimulus set, the a priori classification of conditions (based on the generative geometric procedure) may not correspond to participants’ phenomenological experience of complexity. This underscores the importance of validating structural manipulations against subjective complexity ratings before—rather than only after—the main data collection.

It is important to be explicit about what this failed manipulation does, and does not, allow us to conclude. It does not allow us to conclude that “visual complexity has no effect on attention,” nor does it validate any general claim about complexity as a perceptual or aesthetic construct: what we manipulated and validly confirmed was a specific generative dimension (disordered, small-scale tessellation vs. large-scale bilateral symmetry), and what participants’ complexity ratings tracked evidently diverged from that dimension in the HC condition specifically. Every null or positive result involving the HC condition in this study should therefore be read as a finding about this particular stimulus manipulation and how it was subjectively construed, not as a finding about complexity in general. Conversely, the null eye-tracking and facial-coding results (Section 3.1 and Section 3.5) cannot be cleanly attributed to the complexity manipulation having failed, because the HH-vs-SC contrast—which does not depend on the disputed HC condition and did track the intended symmetry/motif manipulation—was equally null on every oculomotor and facial-coding measure. What can be concluded with reasonable confidence is narrower than our original framing suggested: this specific generative manipulation of structural regularity, whatever exact perceptual dimension it engaged, shifted subjective complexity judgments but did not produce a statistically detectable change in overt gaze or facial engagement under the present design.

### 4.2. Overt Attention and Facial Engagement Were Insensitive to Structural Condition

None of the fixation-based or facial-coding metrics distinguished the three conditions, and, as detailed in Section 2, this design was powered post hoc to detect only medium-to-large effects ($\eta_{p}^{2}\geq 0.114$); the observed effect sizes for every eye-tracking and facial-coding metric fell well below this threshold ($\eta_{p}^{2}=0.003$–0.046 and 0.010–0.028, respectively). Following standard guidance on interpreting non-significant findings [26], we treat this pattern as an absence of detectable differences under the present sample size and measurement precision, not as evidence that visual structure has no effect on overt attention or facial engagement; a true effect in the small-to-medium range cannot be ruled out. Several factors specific to the present design may additionally account for this null pattern. The 6–7 s exposure window, while sufficient to capture initial orienting, may be too brief for global structural properties to shape the overall distribution of fixations or facial engagement, particularly if such effects unfold gradually over longer viewing periods. The use of webcam-based eye tracking and facial coding (approximately 30 Hz sampling) also entails greater spatial, temporal, and signal-to-noise limitations than laboratory-grade systems, which may have obscured comparatively subtle condition effects—reflected in the modest effect sizes observed even for the non-significant contrasts ($\eta_{p}^{2}=0.01$–0.05 for eye-tracking metrics; $\eta_{p}^{2}=0.01$–0.03 for facial-coding metrics). It is also possible that, for abstract geometric patterns lacking semantic anchors, low-level salience (contrast, color, local symmetry) exerts a stronger pull on early fixations than global structural regularity, effectively equalizing scanning and facial-engagement behavior across conditions despite their differing formal properties.

In earlier analyses of this dataset, we additionally argued that the coefficient *K*’s correlation with independent oculomotor indices of focal processing supported the construct validity of the combined measurement approach, and thus indicated that the null condition effects were unlikely to reflect a wholesale failure of measurement. Having corrected those correlations for the non-independence of repeated observations (Section 3.6), we no longer consider this argument well supported: none of the *K*–oculomotor associations survives appropriate correction, and the association with fixation duration reverses sign once repeated-measures structure is accounted for. We therefore do not rely on *K*’s convergent validity to argue that the measurement pipeline was functioning correctly; the case that the null condition effects reflect limited power and precision rather than a broken instrument rests instead on the sensitivity analysis above, together with the platform validation evidence discussed in Section 2.3 [22]. We regard the status of *K* as a valid index of ambient/focal processing in this particular abstract, brief-exposure paradigm as an open question rather than a settled one.

### 4.3. A Fragile, Nominal Association Between Scanpath Entropy and Pleasantness

In our initial, uncorrected analyses, both spatial dispersion and scanpath entropy appeared negatively correlated with pleasantness ratings, which we interpreted as running counter to the exploratory-processing account proposed in the Introduction. Once repeated-measures correlation and correction for multiple comparisons are applied (Section 3.3), the dispersion–pleasantness association is no longer distinguishable from zero, and only the entropy–pleasantness association remains nominally significant ($r_{rm}=-0.23$, p=0.044)—itself not surviving FDR correction across the full exploratory correlation family (q=0.201). We therefore no longer treat this pattern as an established empirical result, but discuss below how it should be interpreted if it reflects a genuine, if weak, effect, while emphasizing that this discussion is now explicitly speculative rather than an interpretation of a confirmed finding.

The negative correlation between scanpath entropy and pleasantness ratings, to the extent it reflects a real effect, runs counter to the exploratory-processing account proposed in the Introduction, in which distributed viewing was expected to accompany more positive aesthetic responses. This pattern also runs counter to a substantial body of literature reporting the opposite association—that more, and more prolonged, looking tends to accompany greater liking rather than less. The gaze cascade effect, for instance, shows that gaze is progressively biased toward the item that will eventually be chosen or rated as more attractive, implicating a positive feedback loop between looking and preference [30]—a pattern independently found to replicate under webcam-based, browser-delivered eye tracking of the same general kind used here [31], suggesting the discrepancy with our own results is unlikely to be a simple artifact of webcam-based measurement per se; in an applied advertising context, increased number and duration of fixations on an advertisement has similarly been found to correlate with more positive evaluations of it [32]. Both of these literatures, however, involve either an explicit choice between competing alternatives (gaze cascade) or naturalistic, semantically rich stimuli (advertisements)—conditions that differ substantially from the single-stimulus, free-viewing paradigm with abstract geometric patterns used here, and in which the mechanism linking gaze to preference (value accumulation toward a decision, or engagement with meaningful content) may not readily apply.

Closer to the present paradigm, single-stimulus free-viewing studies of pleasantness are more mixed. In an eye-tracking study of nature and urban scenes, images rated as more pleasant elicited fewer fixations but longer individual fixations [33], a pattern that, in terms of fixation count at least, aligns with the direction we observed rather than the increased-exploration account. More broadly, reviews of fixation-duration operationalizations in aesthetic and appreciation research caution that longer looking does not unambiguously index greater liking, because processing difficulty—arising from lower visibility, greater clutter, or a harder-to-resolve percept—can independently prolong fixation and viewing time on stimuli that are not necessarily preferred [34]. This is one alternative interpretation worth entertaining, if the entropy–pleasantness association reflects a genuine effect rather than a false positive: broader, less concentrated scanning (higher scanpath entropy) may reflect difficulty resolving a stimulus into a coherent percept—an uncertainty-driven search pattern—whereas preferred stimuli may instead sustain more focal engagement on a small number of privileged regions. This would be consistent with processing-fluency accounts of empirical aesthetics, in which stimuli that are easier to process (and often preferred) are associated with more efficient, less dispersed visual exploration [16], and with Berlyne’s classic proposal that moderate, easily resolved arousal potential—rather than the sheer amount of exploratory activity a stimulus elicits—underlies hedonic value [14].

Reconciling these conflicting literatures is beyond the scope of a single study, but the contrast is informative: gaze-cascade and advertising findings involve either explicit choice or content-rich stimuli where looking plausibly serves information gathering toward a decision or engagement with meaningful features, whereas the present free-viewing task with abstract, non-representational patterns and no choice requirement may instead be dominated by processing-fluency dynamics, in which dispersed, effortful scanning signals a percept that resists easy resolution. Given that our own evidence for this pattern is now nominal at best, we present this as one plausible account to be tested, not as an established finding. Future work directly manipulating task demands (choice vs. passive viewing) and stimulus meaningfulness (abstract vs. representational) within the same paradigm, with a sample size and correlational approach that accounts for repeated measures from the outset, would help establish whether an association of this kind exists and, if so, which boundary conditions govern its sign.

### 4.4. A Tentative Parallel with Dissociations Reported Elsewhere

The pattern found here—a robust effect on subjective complexity judgment alongside no detectable condition effect on fixation-based or facial-coding measures—is at least broadly compatible with a dissociation documented elsewhere between covert or overt attentional signals and explicit evaluative preference, though we emphasize that our own null oculomotor and facial-coding results should be read as an absence of detectable effects under this design (Section 4), not as a demonstrated dissociation in the strong sense. In face perception, covert and overt measures of attention to facial attractiveness have been shown to diverge according to task demands, with reliable oculomotor preferences emerging only under some measurement conditions [18]. In developmental work on symmetry, four-year-old children reliably fixate symmetrical patterns longer than asymmetrical ones yet show no corresponding explicit preference for them, directly dissociating spontaneous visual attention from aesthetic judgment [19]. More broadly, the symmetry-preference literature suggests that the link between fixation behavior and aesthetic evaluation is itself categorically variable, holding for some stimulus domains but not others [6], and that spontaneous oculomotor signatures of structural processing (e.g., axis-aligned scanning of symmetrical patterns) can be similar across classification and evaluation tasks even when explicit preference diverges from perceptual sensitivity [7]. The present results are consistent with an extension of this general pattern to adult observers viewing abstract geometric stimuli—structural properties shifted what people said they preferred (for complexity, robustly so) without producing a statistically detectable signature in either oculomotor or facial-expressive channels under the present design—but given the corrections applied in this revision, we hold this extension more tentatively than in our initial analysis, and present it as a hypothesis the present data are consistent with rather than one they confirm. This raises the possibility that evaluative judgments of harmony and complexity are computed, at least in part, through processes that are not fully indexed by overt gaze allocation or momentary facial affect—a possibility that merits direct investigation with adequately powered samples, repeated-measures-aware analyses from the outset, and converging measures (e.g., pupillometry, EEG) in future work.

### 4.5. Two Contributions of This Study, Held to Different Standards of Evidence

We close the Discussion by returning explicitly to the dual contribution flagged in the Introduction, because the two halves of this study now rest on different standards of evidence and should be weighted accordingly by the reader. The substantive contribution—that structural disorder, as we operationalized it, does not translate straightforwardly into perceived complexity, and instead produced a reversed ordering—is a single-experiment finding from one specific stimulus set and should be treated as a hypothesis for the field to test further (Section 5, item 1), not as a general claim about visual complexity. It is, however, the one primary result in this study that survives our strictest correction for multiple comparisons, and it converges with an independent, purely computational literature indicating that structural regularity is only one of several separable contributors to perceived complexity [13].

The methodological contribution is, in our view, on firmer ground precisely because it does not depend on any single substantive effect being real. Analyzing this dataset surfaced two analytic problems that are neither unique to our data nor, we suspect, unique to this literature: correlating repeated within-participant observations without accounting for their non-independence, and reporting a large family of eye-tracking, facial-coding, and subjective outcomes without a stated multiplicity strategy. Both problems are straightforward to introduce inadvertently in exactly the kind of multi-condition, multi-exemplar, multi-measure webcam-based eye-tracking design that is becoming increasingly common as platforms like RealEye lower the barrier to running such studies remotely and at scale [23,24]. We report, in Section 3 and Section 4, a concrete demonstration of how much a study’s conclusions can change once these two corrections are applied—including the reversal of one association and the loss of significance for several others we had initially interpreted as supporting our measurement approach—together with the specific procedures used (repeated-measures correlation via an ANCOVA-equivalent formulation, and Benjamini–Hochberg FDR correction across the exploratory family designated in Section 2). We offer this worked example, independent of whether the substantive complexity finding itself replicates, as a practical resource for researchers designing or reanalyzing similarly structured studies.

## 5. Conclusions

Controlled variations in visual harmony and complexity, isolated from semantic content, shaped participants’ subjective complexity judgments—the only one of seven primary, family-wise-corrected omnibus tests to survive correction—but did not produce statistically detectable differences in overt attentional allocation, as measured by fixation counts, durations, latencies, and spatial/entropy-based dispersion indices, nor in facial-coding measures of expression and ambient/focal engagement. The complexity manipulation itself did not translate into the intended perceptual ordering of conditions: what we can conclude is narrower than we originally framed it, and concerns this specific generative manipulation rather than visual complexity as a general construct (Section 4). A nominally significant effect on pleasantness ratings, and nominally significant correlations between eye-tracking/facial-coding metrics and subjective ratings—including the coefficient *K*’s associations with independent oculomotor indices, which we had initially read as supporting the validity of our combined measurement approach—did not survive correction for the non-independence of repeated observations and for the number of statistical tests performed, and are reported here as exploratory rather than confirmatory findings. Together, these findings suggest, more tentatively than our initial analysis proposed, that formal visual structure may influence evaluative, higher-order aesthetic responses more readily than early overt attentional or facial-affective deployment, at least under brief, free-viewing conditions with webcam-based measurement—a pattern broadly consistent with, though not decisive evidence for, the dissociation between attention and preference documented in other domains [18,19]. The absence of detectable oculomotor and facial-coding effects should not be read as evidence that visual structure has no effect on attention [26]; rather, it reflects what a post-hoc-determined, webcam-based, brief-exposure design with N=40 was, and was not, equipped to detect.

Based on the specific limitations identified throughout this study, we consolidate the following directions for future research:

1. Independently validate stimulus manipulations before data collection. A dedicated pilot study should collect subjective complexity and harmony ratings for candidate stimuli and use them to select or adjust the final exemplars, rather than relying solely on the a priori generative parameters used here (Section 2 and Section 4).
2. Conduct a prospective power analysis. Future replications should determine the target sample size before data collection, using the effect sizes and repeated-measures correlation structure reported here (Section 3.4 and Section 3.3) rather than relying on a post-hoc sensitivity analysis as this study necessarily did.
3. Increase spatial and temporal measurement precision. Replicating the design with laboratory-grade infrared eye tracking (sub-degree accuracy, ≥250 Hz) would clarify whether the null oculomotor effects reflect a genuine absence of structural modulation or the coarser precision of webcam-based recording (Section 2.3).
4. Extend and vary exposure duration. Systematically comparing brief (<10 s) and extended (e.g., 20–30 s) free-viewing windows within the same design would establish whether global structural effects on gaze require more time to emerge than local salience-driven orienting.
5. Increase the stimulus set and statistical power. A larger item pool (beyond three exemplars per condition) together with a sample size informed by the sensitivity analysis reported here (Section 3.4) would allow detection of the small ($\eta_{p}^{2}<0.114$) effects that the present design could not rule out.
6. Define areas of interest a priori. Region-specific measures (e.g., time to first fixation on the central motif versus peripheral facets) may be more sensitive to the harmony/complexity manipulation than whole-stimulus metrics.
7. Directly test the boundary conditions of the entropy–pleasantness relationship. Manipulating task demands (explicit choice vs. passive free viewing) and stimulus meaningfulness (abstract vs. representational) within a single paradigm would help establish whether the nominal association observed here is genuine and, if so, how it relates to the positive associations reported in gaze-cascade and advertising research (Section 4).
8. Incorporate converging psychophysiological measures. Pupillometry and EEG could help determine whether evaluative judgments of harmony and complexity are computed through processes that are not fully indexed by overt gaze allocation or momentary facial affect.
9. Pre-register the analysis plan, including the correlational approach and multiplicity strategy. Specifying primary versus exploratory outcomes, the repeated-measures correlation method, and the multiple-comparison correction procedure in advance would remove the need for the kind of post-hoc reanalysis undertaken in this revision and would strengthen confidence in whichever pattern of results is obtained.

## Figures and Tables

**Table 1:** Repeated-measures ANOVA results for eye-tracking metrics across the three stimulus conditions (N=40).

Metric	HH *M* (SD)	HC *M* (SD)	SC *M* (SD)	F(2,78)	*p*	$\eta_{p}^{2}$
Number of fixations	24.11 (2.58)	24.16 (2.83)	24.02 (2.21)	0.10	0.902	0.003
TTFF (ms)	44.30 (48.14)	70.80 (102.90)	65.18 (77.38)	1.83	0.167	0.045
Fixation duration (ms)	266.30 (64.84)	266.43 (66.46)	263.64 (59.88)	0.45	0.641	0.011
Heatmap dispersion	32.42 (10.63)	33.46 (11.39)	32.66 (13.44)	0.45	0.637	0.012
Scanpath entropy (norm.)	0.46 (0.10)	0.46 (0.08)	0.45 (0.10)	1.89	0.158	0.046

**Table 2:** Linear mixed-effects model results (participant- and item-level random intercepts, item-level observations, N=40×9). HC and SC estimates are relative to the HH baseline.

Metric	HC − HH (*p*)	SC − HH (*p*)	Omnibus LRT $\chi^{2}\left(2\right)$, *p*
Number of fixations	0.05 (0.870)	−0.08 (0.785)	0.20, 0.907
TTFF (ms)	26.50 (0.109)	20.88 (0.206)	3.67, 0.160
Fixation duration (ms)	0.13 (0.974)	−2.65 (0.513)	0.76, 0.684
Heatmap dispersion	1.05 (0.432)	0.25 (0.852)	0.68, 0.712
Scanpath entropy (norm.)	0.008 (0.442)	−0.010 (0.371)	2.80, 0.247
Pleasantness rating	−0.27 (0.018)	0.12 (0.300)	12.05, 0.002
Complexity rating	−0.45 (0.012)	0.08 (0.675)	8.14, 0.017

**Table 3:** Repeated-measures ANOVA results for facial-coding metrics across the three stimulus conditions (N=40).

Metric	HH *M* (SD)	HC *M* (SD)	SC *M* (SD)	F(2,78)	*p*	$\eta_{p}^{2}$
Neutral (0–1)	0.13 (0.09)	0.12 (0.08)	0.14 (0.10)	1.12	0.333	0.028
Happy (0–1)	0.08 (0.13)	0.08 (0.12)	0.10 (0.14)	0.89	0.415	0.022
Surprise (0–1)	0.02 (0.02)	0.02 (0.02)	0.02 (0.02)	0.72	0.490	0.018
Coefficient *K*	0.25 (0.16)	0.24 (0.14)	0.26 (0.19)	0.40	0.670	0.010

**Table 4:** Coefficient *K* vs. oculomotor indices: naive Pearson correlation (treating the 120 condition-level observations as independent) versus repeated-measures correlation ($r_{rm}$, N=120 observations from k=40 participants, $df_{2}=79$). *q* = Benjamini–Hochberg FDR-adjusted *p*-value across the full 23-test exploratory correlation family.

Metric	Naive *r* (*p*)	$r_{\mathrm{rm}}$	*p*	*q*
Number of fixations	+0.08 (0.402)	+0.30	0.007	0.170
TTFF	−0.38 (<0.001)	−0.19	0.098	0.281
Fixation duration	+0.27 (0.003)	−0.24	0.034	0.193
Heatmap dispersion	+0.21 (0.021)	+0.02	0.879	0.961
Scanpath entropy (norm.)	+0.12 (0.194)	−0.02	0.834	0.961

**Table 5:** Descriptive statistics (mean (SD)) by individual stimulus exemplar (N=40).

Item	Cond.	N Fixations	TTFF (ms)	Fix. Dur. (ms)	Dispersion	Entropy (Norm.)	Pleasantness	Complexity
Img 1_HH	HH	24.43 (3.69)	45.35 (112.29)	263.89 (71.54)	31.08 (11.37)	0.45 (0.13)	3.80 (1.14)	3.73 (1.18)
Img 2_HH	HH	24.10 (3.18)	37.05 (80.64)	260.22 (67.51)	33.25 (10.41)	0.48 (0.10)	3.73 (1.30)	3.85 (1.05)
Img 3_HH	HH	23.80 (3.25)	50.50 (84.63)	274.78 (68.95)	32.91 (15.77)	0.44 (0.14)	3.65 (1.31)	3.58 (1.11)
Img 1_HC	HC	24.05 (3.19)	102.33 (192.49)	269.80 (67.83)	36.13 (21.00)	0.47 (0.11)	3.58 (1.17)	3.27 (1.20)
Img 2_HC	HC	24.57 (3.64)	51.45 (93.11)	262.18 (74.38)	31.76 (14.71)	0.46 (0.13)	3.50 (1.24)	3.12 (1.28)
Img 3_HC	HC	23.85 (3.25)	58.62 (116.31)	267.31 (69.71)	32.49 (10.01)	0.47 (0.10)	3.30 (1.29)	3.40 (1.26)
Img 1_SC	SC	24.05 (2.91)	43.98 (70.00)	262.62 (59.40)	33.67 (16.03)	0.45 (0.12)	3.85 (1.17)	3.48 (1.01)
Img 2_SC	SC	24.40 (2.98)	66.58 (121.31)	263.26 (62.88)	32.56 (16.36)	0.44 (0.11)	3.98 (1.29)	4.12 (1.04)
Img 3_SC	SC	23.62 (2.67)	85.00 (166.02)	265.05 (68.08)	31.77 (13.89)	0.45 (0.12)	3.70 (1.32)	3.77 (1.17)

## Data Availability

The eye-tracking, facial-coding, and survey data generated during the study, together with the derived condition-level metrics, are available from the corresponding author on reasonable request. The geometric stimulus set used in this study, and the analysis code used to compute eye-tracking, facial-coding, and statistical results, are likewise available from the corresponding author on reasonable request.
